# Polygenic Risk Scores for Amyloidosis as Predictors of Early changes in Alzheimer's Disease Endophenotypes

**DOI:** 10.1002/alz70856_101027

**Published:** 2025-12-24

**Authors:** Yuexuan Xu, Min N Qiao, Tamil Iniyan Gunasekaran, Yian Gu, Dolly Reyes‐Dumeyer, Angel Piriz, Danurys Sanchez, Belisa Soriano, Yahaira Franco, Zoraida Dominguez Coronado, Patricia Recio, Diones Rivera Mejia, Martin Medrano, Rafael A. Lantigua, Ramiro Eduardo Rea Reyes, Rachael E. Wilson, Jennifer J. Manly, Adam Brickman, Corinne D. Engelman, Sterling C Johnson, Sanjay Asthana, Badri N. Vardarajan, Richard Mayeux

**Affiliations:** ^1^ Columbia University, New York, NY, USA; ^2^ The Taub Institute for Research on Alzheimer's Disease and the Aging Brain, Columbia University, New York, NY, USA; ^3^ G.H. Sergievsky Center, Columbia University, New York, NY, USA; ^4^ The Gertrude H. Sergievsky Center, College of Physicians and Surgeons, Columbia University, New York, NY, USA; ^5^ Cognitive Neuroscience Division, Columbia University, New York, NY, USA; ^6^ Columbia University Irving Medical Center, New York, NY, USA; ^7^ Department of Neurology, Vagelos College of Physicians and Surgeons, Columbia University, and the New York Presbyterian Hospital, New York, NY, USA; ^8^ Gertrude H. Sergievsky Center, Vagelos College of Physicians & Surgeons, Columbia University, New York, NY, USA; ^9^ Columbia University Medical Center, New York, NY, USA; ^10^ Universidad Pedro Henríquez Urena, Santo Domingo, Dominican Republic; ^11^ Clinica Corominas, Santiago, Dominican Republic; ^12^ Clinica Gregorio Hernandez, Puerta Plata, Dominican Republic; ^13^ CEDIMAT, Santo Domingo, Dominican Republic; ^14^ School of Medicine, Universidad Nacional Pedro Henriquez Ureña, Santo Domingo, Dominican Republic; ^15^ CEDIMAT, Plaza de la Salud, Santo Domingo, Santo Domingo, Dominican Republic; ^16^ Pontificia Universidad Católica Madre y Maestra, Santiago, Santiago, Dominican Republic; ^17^ The Taub Institute for Research on Alzheimer's Disease and the Aging Brain, Vagelos College of Physicians & Surgeons, Columbia University, New York, NY, USA; ^18^ Wisconsin Alzheimer's Institute, University of Wisconsin‐Madison School of Medicine and Public Health, Madison, WI, USA; ^19^ Department of Medicine, University of Wisconsin‐Madison School of Medicine and Public Health, Madison, WI, USA; ^20^ Wisconsin Alzheimer's Institute, University of Wisconsin School of Medicine and Public Health, Madison, WI, USA; ^21^ Taub Institute for Research on Alzheimer's Disease and the Aging Brain, Vagelos College of Physicians and Surgeons, Columbia University, New York, NY, USA; ^22^ Department of Neurology, Vagelos College of Physicians and Surgeons, Columbia University, New York Presbyterian Hospital, New York, NY, USA; ^23^ G. H. Sergievsky Center, Vagelos College of Physicians and Surgeons, Columbia University, New York, NY, USA; ^24^ Taub Institute for Research on Alzheimer's Disease and the Aging Brain, Vagelos College of Physicians and Surgeons, Columbia University, New York, NY, USA; ^25^ Department of Neurology, Vagelos College of Physicians and Surgeons, Columbia University, New York, NY, USA; ^26^ Department of Population Health Sciences, University of Wisconsin School of Medicine and Public Health, Madison, WI, USA; ^27^ Wisconsin Alzheimer's Disease Research Center, University of Wisconsin School of Medicine and Public Health, Madison, WI, USA; ^28^ Department of Neurology, Columbia University, New York, NY, USA

## Abstract

**Background:**

Early identification of individuals at risk for Alzheimer's disease (AD) is critical for effective intervention. Amyloid deposition, a hallmark of AD pathology, offers insights into genetic factors driving disease progression. While the *APOE* genotype is a major contributor, the role of other risk variants in amyloid deposition and early AD endophenotypes remains unclear. This study aims to (1) identify amyloid polygenic risk scores (PRS) that best predict amyloid burden in Europeans and Hispanics beyond *APOE*, (2) compare the amyloid PRS with *APOE*, an AD risk PRS, and a tau PET PRS, and (3) validate these PRSs against AD endophenotypes in non‐demented older adults.

**Method:**

Among Europeans, amyloid PRSs were developed using GWAS of amyloid PET, plasma *p*‐tau181, and CSF *p*‐tau181, along with separate PRSs for AD risk and tau PET burden. For Hispanics, amyloid PRSs were derived using GWAS of plasma *p*‐tau181 and the GAUDI method, which incorporates individual‐level plasma *p*‐tau181 and local ancestry among admixed population. Multi‐ancestry PRSs were constructed using PRS‐CSx from GWAS of plasma *p*‐tau181 and AD across ancestries. After parameter tunning, all PRSs were tested in two datasets (515 Hispanics and 1,120 Europeans) using linear and mixed‐effects models to evaluate cross‐sectional and longitudinal associations with plasma *p*‐tau181, *p*‐tau217, Aβ42, Aβ42/Aβ40, GFAP, NfL, and global cognition.

**Result:**

Among Europeans, the *APOE*‐ε4 allele explains 3.6%–6% of the variance in *p*‐tau181 (β=0.071, 95%CI: 0.034–0.108) and *p*‐tau217 (β=0.127, 95%CI: 0.101–0.152). PRSs derived from amyloid‐PET GWAS explain 3%–4% of the variance in *p*‐tau181 (β=0.039, 95%CI:0.018–0.060) and *p*‐tau217 (β=0.036, 95%CI: 0.022–0.051). Among Hispanics, the *APOE*‐ε4 allele explains 1% of the variance in *p*‐tau181 (β=0.057, 95%CI: 0.004–0.109), while GAUDI PRSs explain 1.5% (β=0.032, 95%CI: 0.009–0.054). Among non‐demented older adults, in Europeans, amyloid‐PET PRSs are associated with plasma Aβ42, Aβ42/Aβ40, GFAP, NfL, and global cognition, while GAUDI PRSs show similar associations in Hispanics. Other PRSs derived from AD or tau GWAS or multi‐ancestry PRS show weaker or no associations in both ancestries.

**Conclusion:**

Amyloid PRSs show promise as predictive tools for early AD pathology in asymptomatic individuals among Europeans and Caribbean Hispanics.

